# Supplementary material for: Are carbon emissions trading and green financial instruments synergistic? -Comprehensive quantitative research based on content analysis
Source: PLoS One. 2024 Mar 7;19(3):e0298601. doi: 10.1371/journal.pone.0298601 (PMC10919601; doi:10.1371/journal.pone.0298601)
Supplement: S1 Data — (ZIP) [file pone.0298601.s001.zip › Data/地级市Z_F工作报告.zip!/╝¬┴╓/░╫╔╜╩╨/└┤╫╘╣1⁄2╓┌║┼í╛┬φ┐╦╩2╛▌═°í┐_2010.docx]

各位代表：

　　我代表白山市人民政府，向大会作政府工作报告，请予审议。并请各位政协委员和其他列席人员提出意见和建议。

　　一、凝心聚力，攻坚克难，2009年白山经济社会发展跃上新台阶

　　过去的一年，在省委、省政府和市委的正确领导下，全市上下积极应对国际金融危机和世界经济严重衰退带来的不利影响，坚持把“保增长、保民生、保稳定”作为全部工作的主题，坚定信心，抢抓机遇，凝心聚力，克难求进，全市经济社会呈现出速度加快、效益提高、后劲增强、民生改善的新局面，较好地完成了市六届人大二次会议确定的目标任务。

　　——国民经济持续较快增长，综合实力进一步增强。预计全年实现地区生产总值370亿元，增长21%。完成全口径财政收入36.4亿元、地方级财政收入19.8亿元，分别增长20.8%和26.9%。财政支出76.4亿元，增长31%。工业经济快速增长。预计全年完成规模以上工业增加值170亿元，增长27%，实现净利润13亿元。规模以上企业发展到370户，新增41户。实现新产品产值31.2亿元，增长75.3%。完成技改投资125亿元，增长46.3%。“施慧达”、“地王”牌商标被命名为中国驰名商标。万元工业增加值能耗下降5%，主要污染物化学需氧量和二氧化硫排放量控制在省定指标以内。农村经济全面发展。预计全年实现农林牧渔业总产值62.8亿元，增长9.7%。新增20个千亩以上产业化基地，全市农产品加工企业发展到441户，其中省级以上龙头企业30户。开展了“百村万户升级”工程，为1.2万农户发放家庭经济项目贷款4.3亿元。发放政策性惠农补助和补贴5696万元。第三产业规模不断壮大。成功举办了首届“长白山松花江鸭绿江旅游节”、“松花石文化节”和“第23届人参节”。预计全年实现第三产业增加值121亿元，增长19%。全年接待国内游客283万人次，旅游总收入22.7亿元，分别增长20%和31.6%。实现社会消费品零售总额117亿元，增长21.4 %。项目建设扎实推进。预计全年完成固定资产投资410亿元，增长36.6%。开工建设3000万元以上项目429个，其中亿元以上项目213个。八宝工业园矿井改造、东圣焦化6万吨甲醇等116个项目竣工投产。长白山国际旅游度假区、华能煤矸石发电等330个续建和新开工项目进展顺利。赤松核电站、粉煤灰综合利用、浑江电厂六期扩建等重大项目的前期工作取得突破性进展。有230个项目列入中央扩大内需投资计划，争取中央新增投资7.98亿元。

　　——全方位深化改革开放，对外开放水平进一步提高。各项改革扎实推进。完成了市县两级政府机构改革，市级部门由37个精简到32个。加快政务公开建设，实行重点项目代办制、进厅部门领导带班制，建立重点项目审批绿色通道，即办件数量提高到70%，压缩审批时限83%。集体林权制度改革全面启动，林权矛盾纠纷调处工作走在全省前列。基本完成了国有企业和厂办大集体企业改制。35户国有粮食购销企业产权制度改革全面完成。农村土地承包确权和土地流转管理服务试点工作进展顺利。招商引资取得新成效。深入开展招商引资年活动，重点实施产业推进组招商、委托代理招商和挂职招商，积极赴长三角、珠三角、环渤海及日本、香港等国家和地区进行重点招商，组织参加了东博会、深洽会等重要经贸活动。引进了中国华能、大连万达、天津天士力、上海大山合等一批战略投资者。预计全年引进市外资金193亿元，增长40%。实际利用外资1.1亿美元，增长19.7%；完成外贸进出口总额1.65亿美元，下降23%。

　　——加强城市建设和管理，综合承载功能进一步提升。城市建设取得新突破。市区完成投资24亿元，全力实施了畅、绿、净、亮、美工程。向江大桥建设工程进展顺利，彩虹桥加固改造、白山路人行天桥、三个城区出口改造和31条道路修建工程全部竣工。对城市广场、公园、铁路沿线、浑江沿岸进行了绿化改造，新植树木24万株，新增绿地面积50万平方米。基本完成了污水处理厂主干网和污水管网铺设任务。浑江大街、长白山大街等主要街路路灯改造工程和广电大厦、地旺商厦等17座高层亮化工程全面完工。满族风情园、江源公园投入使用。“城市管理年”活动成效明显。通过开展环境卫生、交通秩序等七项专项整治活动，基本解决了市中心区占道经营、城市脏乱差等问题，人居环境明显改善。鸭绿江干流、松花江出境水质均保持国家二类水体标准，全年空气优良级别天数达到312天。我市被评为中国十大生态旅游城市。交通建设步伐加快。全市预计完成交通建设投资 39.7亿元。环长白山旅游公路、市区南北连接线竣工通车。三道沟至集安界边防公路、靖宇至松江河铁路开工建设。营城子至松江河高速公路、东岗至长白公路续建工程顺利实施。新修农村公路550公里。靖宇至新胜林场一级公路、鹤大高速公路市区连接线及互通立交桥等项目前期工作取得突破性进展。

　　——社会事业不断发展，人民生活进一步改善。各项社会事业全面推进。市急救中心投入使用，全民健身活动中心扩建和儿童福利院、残疾人综合康复就业服务中心主体工程完工。完成34个卫生服务中心、69个乡镇卫生院改造及设备更新。广播电视“村村通”工程、数字电视转换和网络双向化进展顺利。投资1.3亿元改扩建校舍13万平方米。全部免除了11万名城乡义务教育阶段学生学杂费。公共卫生体系不断完善，新农合参合率达到86.9%，年人均筹资标准提高到100元。甲型H1N1流感、手足口病等传染性疾病防控工作取得成效。承办了吉林省第五届社区体育健身大会并连续四年获得全省第一名的好成绩。长白山满族文化博物馆正式开馆。我市被命名为中国松花砚文化产业基地，长白山奇石文化城投入使用。依法加强民族宗教事务管理。八道江、抚松、临江被列入国家兴边富民重点县。双拥工作扎实推进，我市荣获全国军转干部安置工作先进单位。全面落实人口和计划生育政策，人口自然增长率控制在1.07‰。成功承办了国家科技部 “科技列车长白山行”活动。有 34个项目列入国家、省级科技发展计划。精神文明建设成果丰硕，涌现出全国“十大政法英杰”赵春芳、“全国见义勇为英雄”姜志宝、“全国道德模范”朱玉林、“全国自强模范”于春龙等一批先进典型。我市荣获第六届全国见义勇为城市奖。人民生活明显改善。预计城镇居民人均可支配收入15415元，农民人均纯收入5588元，分别增长14%和12%。全年新增就业4万人，下岗失业人员再就业2万人，全面解决了“零就业家庭”就业问题，城镇登记失业率控制在3.9%以内，统筹城乡就业试点和小额担保贷款工作走在全省前列。去年承诺的10件民生实事，城市棚户区、煤矿棚户区、沉陷区综合治理和廉租房建设全部完成。西北岔第二水源地开工建设，实现“五通一平”。完成103处农村安全饮水工程，解决了4.9万人的饮水问题。市区新增供热能力60万平方米、新增燃气用户1.2万户。碱厂沟河、月牙河低洼内涝综合治理工程全面启动，完成投资5300万元；浑江干流青沟子河口至木锨沟河段浆砌石护岸工程全部完工。异地新建市中医院完成一期工程。松江河火车站改造工程开工建设，信号楼主体框架已经完成。城镇低保标准由月人均160元提高到225元，农村低保标准由年人均540元提高到760元，退休人员养老金月人均标准由822元提高到958元。浑江大桥新建工程，完成桥墩基础建设，没有完成主体工程。市区二次供水泵站和支线管网改造工程，由于调整规划设计等原因没有完成。对没有完成的实事，将在今年予以解决。社会保障和救助体系进一步完善。城镇职工基本医疗保险最高支付限额标准由2.8万元提高到3.5万元。4278名国有破产企业退休人员纳入医保，医疗保险参保人数达到83.5万人。参加失业保险人数达到11.4万人，发放失业金 5288万元。发放养老保险金10.3亿元。工伤、生育保险参保人数分别达到16.5万人和17万人。建立医疗二次救助机制，筹集大病医疗救助资金2340万元，救助城市低保对象11700人次。投资5.1亿元完成了11794户农村泥草房和危旧房改造。“平安白山”创建活动成果显著。妥善处理各类群体性事件120起8927人次，排查各类矛盾纠纷2300件，380件信访疑难案件得到有效解决，信访积案化解率达到83.2%。深入开展打黑除恶、命案攻坚等12项专项严打整治活动，综合破案率全省领先。加强边境管控，保持了边境安全稳定。扎实推进邻里守望、社区和农村警务室建设，社会治安防控体系进一步完善。切实加强煤矿、非煤矿山、危险化学品、食品药品、道路交通、消防等专项安全整治，安全生产形势总体稳定，事故发生率同比下降19.9%。加强森林管护，实现连续30年无重大森林火灾。应急管理体系进一步完善，应对突发事件能力明显增强。

　　——全面打造阳光政务，服务型政府建设进一步加强。深入开展学习实践科学发展观活动，全面加强政府机关建设，政府工作效率和服务能力明显提升。办理人大代表议案和政协委员提案139件，办结率达到100%。进一步实施了市长电话值班、市政府领导接访约访下访、政务信息公开、公开征集民意、人民群众监督五项制度，受理各类投诉20347件，办复率在95%以上，信访案件总量同比下降29.8%，上访群众人数同比下降32.1%，我市荣获2009年中国城市管理进步奖。认真开展民主评议政行风、软环境活动，完善听证制度，加大教育收费、医药购销等重点领域的纠风专项治理和涉软案件查处力度，经济发展软环境明显改善。

　　各位代表，2009年是新世纪以来白山经济社会发展面临困难与挑战最为严峻的一年，也是白山应对新挑战、取得新成果、实现新突破的一年。这是省委、省政府和市委统揽全局、正确领导的结果，是市人大、市政协和社会各界有效监督、鼎力支持的结果，是全市各族人民群策群力、拼搏实干的结果。在此，我代表市政府，向全市各族人民，向驻白山的中省属企事业单位、人民解放军指战员、武警部队官兵、公安司法干警，向各民主党派、工商联、人民团体、无党派人士、离退休老同志，向关心和支持白山发展的各界朋友，致以崇高的敬意和衷心的感谢！

　　在看到成绩的同时，我们也清醒地认识到，工作中还存在一些问题和不足。主要是：超前谋划项目和招商引资力度不够，具有较好市场前景和较强拉动作用的大项目偏少。城市建设和管理还存在许多薄弱环节，一些公共设施建设还相对滞后。区域经济发展速度不快，城乡居民收入水平不高，部分群众生活仍然比较困难。政府职能需要进一步转变，公共服务能力和行政执行力有待进一步提升，发展环境尚需进一步优化。对此，我们要在今后的工作中采取有效措施，认真加以解决。

　　二、抢抓机遇，乘势快上，全力推进2010年经济社会更好更快发展

　　今年是全面实现“十一五”规划目标、科学谋划“十二五”规划的关键之年，也是经济发展面临严峻挑战、蕴含重大机遇的一年。当前，世界主要经济体经济出现好转，一些新兴经济体和发展中国家经济增速加快。国内经济回升向好的基础逐步稳固，市场信心明显增强，扩大内需和改善民生的政策效应进一步显现。国家将继续实施积极的财政政策和适度宽松的货币政策，并着力提高政策的针对性和灵活性，特别是新一轮振兴东北老工业基地和长吉图开发开放战略的实施，为我们优化经济结构、加快经济转型注入了新的动力和活力。从我们自身发展看，近年来我们抢抓机遇、加大投入，一批重点项目和重大基础设施建设不断推进，一批产业扩能和技改项目相继完成。这都为我们做好今年工作奠定了坚实基础。但我们也要看到，国际经济运行中不稳定因素依然很多，国内经济增长的内在动力仍然不足，经济运行面临许多挑战。国际金融危机对我市经济发展的影响还在继续，工业经济仍会面临资源型企业生产艰难、外向型企业市场萎靡、企业效益不佳以及财政收入压力增大等诸多不利因素。为此，我们既要坚定必胜信心，又要增强忧患意识，抢抓机遇，趋利避害，牢牢把握科学发展的主动权，努力保持经济社会平稳快速发展。

　　按照市委五届九次全会的部署，今年政府工作的总体要求是：深入贯彻落实党的十七届四中全会和中央经济工作会议精神，努力践行科学发展观，坚持生态立市、工业强市，以富民强市为目标，以保持经济平稳快速发展为首要任务，以推动经济发展方式转变和经济结构调整为主攻方向，做大做强支柱产业，发展壮大特色产业，大力培育新兴产业，不断提升对外开放水平和自主创新能力，全力推进项目建设、招商引资，全面发展社会各项事业，努力建设富裕开放、文明和谐的新白山。

　　综合考虑各方面因素，全市经济社会发展的主要预期目标是：地区生产总值增长20%以上，全口径财政收入增长20%以上，地方级财政收入增长22%以上，全社会固定资产投资增长35%以上，城镇居民人均可支配收入、农民人均纯收入分别增长12%和11.5%，城镇登记失业率、人口自然增长率分别控制在4.6%和3.9‰以内。万元地区生产总值能耗和主要污染物排放总量控制在省定指标以内。突出抓好以下八项工作。

　　（一）坚持投资拉动，突出项目建设，着力推进产业优化升级。

　　大力发展绿色经济、循环经济和低碳经济，谋划抢上一批技术含量高、产业关联度强的大项目，推进经济结构调整和产业优化升级。

　　依托项目开发推进产业升级。围绕做大做强能源、矿产冶金、旅游三大支柱产业，抓紧谋划新上一批投资额度大、带动能力强、产业关联高的大项目。要在加快煤矸石发电、赤松核电站建设的同时，积极推进浑江电厂六期前期工作，加大煤炭和石油天然气等资源的勘探开发力度，打造东北重要的能源基地。要重点围绕硅藻土、镁、硅、铁、钴、金等优势矿产，延伸产业链条，重点抓好镁工业园、硅藻土工业园和临江铸造工业园等特色园区建设，打造吉林省重要的矿产冶金基地。要以长白山、松花江和鸭绿江等优势资源为依托，加快以长白山国际旅游度假区为龙头的旅游项目建设和功能提升，进一步整合自然、人文旅游资源，打造集生态观光、运动休闲、养生保健、会议论坛等高层次旅游产品为主的长白山国际旅游品牌，尽快把旅游业培育成我市新的支柱产业。围绕发展壮大矿泉水、人参、医药、林产品加工、特色农产品五大特色产业，大力培育新材料、现代物流两大新兴产业，坚持引进增量与扩大存量并举，通过技术改造和新产品开发，最大限度释放产能，不断提升产业的规模优势、品牌优势、集群优势和竞争优势，推进接续替代产业的发展和经济转型。坚持把节能减排、节本增效和提升产品档次、实施名牌战略作为产业升级的切入点，支持开发节能、环保技术，大力发展低碳经济、循环经济，不断提升产业层次和市场竞争力。

　　全力以赴抢抓项目建设。全年完成固定资产投资550亿元，其中工业投资要占全社会投资总额的60%以上。计划安排3000万元以上重点项目470项，完成投资380亿元，其中亿元以上项目320项。千方百计推进华能煤矸石发电、施慧达药业技改等190个续建项目，天安镁合金一期工程、天元公司催化剂等120个项目竣工投产。千方百计落实开工条件，加快金刚水泥生产基地、百万吨焦化等194个新项目开工建设。加快推进20个投资10亿元以上重大项目建设，其中赤松核电站项目完成部分大件设备运输路、5公里进厂路及“四通一平”工程，长白山国际旅游度假区北区部分项目完工、南区全面开工建设，煤矸石发电项目主体厂房封闭并进行设备安装。

　　不断完善项目建设推进机制。加强重点项目信息管理平台建设，完善项目建设的指挥系统、组织系统和监督系统，对全市投资3000万元以上项目建立档案，实施项目形象的动态分析管理。继续实行项目领导责任制、全程跟踪制、全程代办制，完善项目一站式审批，提高重大项目组织推进能力，在土地征用、施工组织、审批手续等方面提供高效服务。

　　（二）坚持工业强市，突出提速增效，着力保持工业经济较快增长。

　　全面实施工业产业跃升计划，以“保增长、调结构、增活力、促融合”为主题，努力扩大增量、盘活存量。全市规模以上工业增加值增长25%，实现净利润13亿元以上。

　　扶优促强做大企业。加强工业经济运行的监控和调度，加大煤、电、油、运、资金等生产要素的组织协调力度，促进重点企业最大限度释放产能、达产达效。加快存量企业技术改造、产品研发和扩大规模，重点扶持东圣焦化、金刚水泥、琦祥纸业、泉阳泉饮品公司等100户高成长性的企业，促其尽快成长壮大为工业经济的骨干。加快小企业的生成和发展，尽快形成新的经济增长点，新纳入规模以上统计口径企业60户。鼓励支持中省属企业上项目、扩产能，发挥规模效应。继续实施技能型人才培养、万名小老板创业和千名企业家培训工程，积极引导企业加强管理，降低成本，提高市场竞争力。加大科技投入，全年完成技改投资 154亿元，增长23%，实现新产品产值 46亿元，增长47%。申报省级以上科技发展项目20项。

　　集中精力做大园区。坚持差异化、特色化发展，促进开发区和特色园区布局合理、产业集群、用地集约，提高投资强度和产出效益。长白山生态科技健康产业园完成污水处理厂及主干道等部分基础配套设施建设。包装工业园完成规划拆迁，琦祥纸业三期工程、5000万平方米包装箱项目开工建设。完成镁工业园二次输变电、园区道路等基础设施建设，开工建设天安镁业二期工程。力争天士力20万吨小分子水、长白山天泉30万吨矿泉饮品年内投产，启动未来趋势集团200万吨矿泉水项目建设，加快推进农夫山泉、娃哈哈扩能改造。硅藻土园区全面完成道路、桥梁、供水、排水等基础设施建设，5000吨纳米级二氧化硅、高档涂料等项目开工建设。

　　着力拓宽融资渠道。进一步加强与省级六大商业银行、国家开发银行的战略合作，推动农村信用社向农村商业银行过渡，增强金融机构对地方经济支持力度。完善中小企业信用担保体系，提高市县两级中小企业担保公司担保能力，鼓励自然人、企业法人和其它社会组织组建担保公司和小额贷款公司。成立多功能企业产权交易中心，为中小企业提供便捷融资服务。积极做好企业上市工作，加快喜丰公司、施慧达药业等企业上市培训，力争东锋有色金属公司在国内创业板上市。

　　（三）坚持发挥特色，突出增收致富，着力推进新农村建设。

　　更加注重农业增效、农民增收和农村环境改善，加快现代特色农业发展。实现农业总产值71亿元，增长10%。

　　大力推进农业产业化经营。坚持标准化生产、规模化经营，重点抓好参、蛙、菌、药、菜等特色农产品基地建设，建设“一村一品”专业乡镇11个、专业村60个，全市特产标准化生产率达到55%以上。实施食用菌富民工程，每个县（市）区扶持2个村、200个农户，每户新增食用菌1万袋。严格执行人参产业发展规划和市场准入标准，建设人参精深加工产业园，人参GAP种植面积达到2000万平方米。大力发展特色畜牧业，建成20个长白山特种野猪规模饲养场，饲养总量达到10万头，建设全国最大的特种野猪养殖加工基地。实施“百村万户升级”工程，为全市200个村、2万个农户发放小额贷款5亿元以上。努力建成国家级农产品质量检验中心，新增有机、绿色、无公害农产品认证50个。重点推进万良长白山人参市场二期扩建、长春大明人参综合开发等25个农业产业化项目，完成投资10亿元以上。积极扶持大山合食用菌、宏久参业、利生源等一批龙头企业做大做强，省级以上龙头企业发展到35户。

　　扎实推进新农村建设。继续抓好农村水、电、路、气、房等基础设施建设，改造农村公路320公里、农村电网183公里，新建100个村级农村信息服务站。加强公路、铁路、边境和旅游沿线村屯卫生环境整治，150个村完成“四清四改”。加快松江河等9个“百镇建设工程”重点镇建设。组织实施“百村示范、村村提升”建设工程，新建18个高标准示范村、20个整体推进村。

　　加快林业改革与发展。上半年完成集体林权制度主体改革任务，探索推进森林采伐分类管理、林权流转等配套改革。启动地方国有林场改革试点。全面加强林业生态建设、资源保护和森林防火工作，完成“三北四期防护林”、退耕还林、日元贷款等生态造林25万亩。

　　（四）坚持搞活商贸流通，突出旅游业发展，着力提升现代服务业水平。

　　以创建中国优秀旅游城市为目标，努力构建以旅游为牵导的现代服务业体系。全年实现第三产业增加值145亿元，增长20%。

　　大力发展旅游业。完成全市旅游总体规划修编，做好重点景区景点详规，整合长白山、松花江、鸭绿江三大旅游品牌资源和区域文化内涵，宣传推介以生态旅游、度假旅游、文化旅游为核心的精品旅游线路，全力打造“生态美谷·白山市”旅游品牌。抓好旅游项目建设，重点加快长白山国际旅游度假区、仙人桥温泉旅游度假区等一批项目建设，做好鸭绿江沿线六个旅游综合服务区规划建设。雨润长白山国际大酒店、临江国际大酒店投入使用，进一步提高旅游承载能力。积极开发松花石、长白玉、黑陶等具有长白山特色的旅游纪念品。继续办好特色旅游节庆活动，不断扩大白山在国内外旅游市场的影响力，推动我市旅游产业快速发展。全年争取接待游客310万人次，实现旅游总收入31亿元，分别增长10%和36%。

　　着力完善商贸流通体系。加快老营火车站内陆港建设，靖宇物流产业园区和江源嘉诚物流中心开工建设，积极筹建江北汽贸园区和废旧金属回收交易园区，推进星泰市场、长白山山货市场异地新建工程。着力构建市区便民服务体系，抓好“菜篮子”网点建设。完善农村商品流通网络，争取建成1000户标准化农家店，提升农村综合流通能力。进一步做好家电、汽车摩托车下乡工作，继续实施家电和汽车以旧换新政策，扩大居民消费需求。积极发展资产评估、咨询服务等中介机构和金融、保险、信息等新兴服务业。全年实现社会消费品零售总额143亿元，增长22%。

　　（五）坚持合作开发，突出招商引资，着力扩大对外开放。

　　进一步加大招商引资和对外合作力度，以开放促发展，努力构建全方位、宽领域的对外开放新格局。

　　强力推进招商引资。全年引进市外资金240亿元，增长25%。着力抓好产业推进组招商。突出绿色食品、矿泉水、人参、旅游等产业优势，每个产业谋划包装10个3000万元以上重点项目，落实2个以上招商项目。着力抓好重点区域招商。以欧美、东南亚及京津鲁、长三角、珠三角等地为重点，积极开展高层次、小分队、专业化招商，不断拓宽合作领域，巩固招商成果，提高履约率。继续实施挂职代理招商，跟踪落实区域内招商在谈项目，并进一步设立招商网点，聘请招商顾问，实行委托代理招商。积极参加省组织的东博会等经贸活动，办好旅游节、矿泉节、人参节等节庆活动，促成更多的项目和资本落户白山。

　　努力扩大对外开放。全市完成外贸进出口总额1.9亿美元，增长15%。重点扶持金隆木业、丽美坚、创萌参业等骨干企业扩大出口。加大国际市场开拓力度，组织50户以上企业参加国内重要展会，推动30户以上企业参加国际展会，外贸进出口经营权企业力争达到300户。做好长白口岸升级验收工作，争取启动建设临江中朝大桥和长白县中朝互市贸易区。积极参与长吉图、东北东部地区合作，促进合作领域向大规模、深层次、全方位发展，提升白山整体竞争力。

　　（六）坚持建管并重，突出完善功能，着力提升城市品位。

　　坚持高起点规划、高标准建设、高效能管理，不断增强城市的承载力、聚集力和辐射力。

　　严格执行规划。完成城市总体规划编制，加快中心城区控制性详细规划编制，力争覆盖率达到60%以上。编制好各个专项规划，重点完成浑江大街沿线景观、滨江景观、市政基础、工业园区的规划编制和碱厂沟河、气象山东侧概念性规划编制。严格规划审批监察，对无控制性详规、不符合城市总体规划的项目一律不予审批。

　　加快城市建设。重点实施市区道路畅通、绿化美化、净化、亮化、旧城改造五大工程。向江大桥、老营沟桥竣工通车，江源大桥开工建设，新建续建博雅路、文福路、富民街等36条道路。完成长白山广场、翠柏广场、南岭广场建设。加快东兴街、浑江大街、月园绿化改造，新增绿化面积5.5万平方米。污水处理厂和城区污水管网工程投入使用，新建移动式垃圾中转站4座、有机处理厂1座、分拣式果皮箱400个，新改建公厕40座。对市区出口、希望广场、文化广场、堤滨园、北山公园实施亮化，完成市区15栋高层建筑楼体亮化工程。加快合兴大厦购物中心、隆府大厦、福泰大厦建设及周边环境改造。

　　加强城市管理。继续深入开展“城市管理年”活动，开展城市管理进学校、进社区、进单位、进机关活动，实行网格化管理，形成全民合力推进城市管理机制。扩大园林、小区绿化面积，增设主要街路、物业小区环卫设施，加强市政设施维护，营造良好的公共活动空间。开展专项整治活动，集中治理红旗贸易城、金河、金街露天市场脏、乱、差问题，整顿市区洗车、物流等行业占道经营和机动车乱停乱放、违章行驶等行为，解决重点路段交通拥堵问题。加强物业管理和服务，着力解决楼道小广告、脏水外溢、烟尘污染、噪声扰民等群众反映强烈的热点难点问题。

　　加快交通建设。总投资79.8亿元，新建靖宇至新胜林场一级公路、靖宇国防公路，续建营城子至松江河高速公路、东岗至长白公路、三道沟至集安界边防公路、靖宇至松江河铁路，力争开工建设鹤大线抚松至小沟岭段、鹤大高速公路市区连接线及市区互通立交桥。积极推进长春经白山至临江、松江河至长白高速公路和铁路的前期工作。

　　（七）坚持开源节流，突出财税征管，着力壮大地方财政实力。

　　坚持生财、聚财、理财并重，进一步完善财政体制，推进政府财政向民生财政、公共财政转变。

　　力保财政收入增长。深入挖掘税源，积极扶持新项目投产达效，骨干企业、税源型企业扩产增效，保证税收增长。完善国有资产监管体制和营运平台，实现国有资产保值增值，增加财政收入。继续加大综合治税力度，完善收入异动预警机制，加强重点税种、重点行业和企业以及新兴行业税源征管，严厉打击偷漏税等违法行为，确保应收尽收。统筹政府非税收入，严格执行“收支两条线”。积极争取上级转移支付资金和项目建设资金，最大限度增加可用财力。

　　优化财政支出结构。坚持保重点、保民生、保运转、保发展，新增财力的70%用于民生和公用事业。优化财政资源配置，不断完善公共财政体系。开展财政监督管理年活动，实现科学化、精细化管理。强化预算刚性约束，保证重点支出，严控一般性支出。

　　（八）坚持协调发展，突出民生保障，着力构建和谐白山。

　　加快推进社会事业建设，扩大公共服务。着力改善民生，让改革发展的成果更多地惠及人民群众。

　　大力发展社会各项事业。全面落实免除城乡义务教育学杂费政策，推动教育均衡发展。大力发展高等教育和职业教育，加快建设长白山职业技术学院，完成市职业技术学校实训楼和县级职教中心建设。进一步深化医药卫生体制改革，大力开展城市社区卫生服务和新型农村合作医疗，提高城乡医疗卫生服务水平。完成中医院建设，启动市精神病医院新建工程。全面加强公共卫生管理，有效防控甲型H1N1流感等重大传染病。积极参加省十六届运动会并争取更好的成绩。贯彻落实民族宗教政策，促进民族地区经济社会发展。继续稳定低生育水平，提高出生人口质量。全面推进公共文化服务体系建设，提高人民群众文化素养。

　　切实保障和改善民生。加快完善社会保障体系，抓好依法破产企业退休人员参保工作，进一步提高社保补贴范围，提高失业保险标准，实现工伤保险市级统筹、城镇居民医疗保险全覆盖。全年新增养老保险5000人、工伤保险 5000人、生育保险 1万人。城镇职工大病统筹最高报销额度由15万元提高到17万元。城市低保月保障标准由225元提高到255元，农村低保标准由年人均760元提高到960元。全面加强城乡社会救助体系建设，认真开展困难群体专项救助。市儿童福利院、残疾人综合康复就业服务中心年内投入使用。筹建市救灾物质储备库，切实提高防灾救灾应急能力。

　　全力维护社会和谐稳定。深入开展平安白山创建活动，加强社会治安综合治理，进一步完善大案攻坚严打联动机制，强化以网格化巡逻为主体的治安防控体系建设。继续保持高压严打态势，组织开展好“打黑除恶”、“网上追逃”等专项斗争，保障人民群众生命财产安全。不断完善阳光政务“五项制度”，集中精力处理好一批信访积案、难案。高度重视安全生产，加强煤矿、非煤矿山、食品药品、道路交通、消防等公共安全专项整治，坚决遏制重特大事故发生。健全公共安全、自然灾害等社会预警体系和应急救援机制，提高各级政府处置突发公共事件能力。不断完善党政军警民合力固边联动机制，确保边境地区安全稳定。

　　努力办好10件民生实事。一是千方百计稳定和扩大就业，全年新增城镇就业3.4万人，免费培训下岗失业人员1万人。二是投资9.3亿元，完成城市棚户区29万平方米、煤矿棚户区43万平方米住宅楼建设，安置居民1万户。南岭沉陷区9.2万平方米住宅楼交付使用。新建廉租住房5万平方米。三是新建集中供热管网3公里、热力站4座，完成管网三级改造任务。新增管道燃气用户1万户。四是投资7300万元，完成市区污水处理厂主体工程。五是投资1.67亿元，完成西北岔第二水源地大坝工程及部分配套设施。完成市区67座供水泵站、3万户供水支线管网改造工程。新建36处农村安全饮水工程，解决3.5万人饮水难问题。六是投资4000万元，完成浑江干流南岸部分堤防改造工程。七是投资1.5亿元，完成碱厂沟河、月牙河综合治理部分改造工程。八是投资2.2亿元，改造边境泥草房3803户、农村危旧房1129户。九是投资3500万元，异地新建市殡仪馆。十是松江河火车站年底前投入使用。浑江大桥主体工程完工。

　　三、务实重行，奋发有为，切实加强政府能力建设

　　新的形势和任务，对政府工作提出了更高要求，我们必须振奋精神，抢抓机遇，奋勇争先，坚定发展信心不动摇，认定发展思路不改变，咬住发展目标不放松，以新思维谋划新发展，以新举措取得新突破。

　　振奋精神，谋求更大作为，增强带动力。各级政府领导干部要多学习、多探索，认真领会落实中央各项保增长和振兴东北老工业基地的政策措施，对一些深层次的问题深入调研、科学分析，真正找准做好经济工作的切入点，进一步增强工作的前瞻性和针对性。超前谋划“十二五”发展蓝图，在振兴东北老工业基地和长吉图开发开放战略中谋求更大的作为，以更高的目标定位、更高的工作标准，顽强拼搏，争创一流，在困难中大干、在大干中赶超、在赶超中实现预期目标，带动全社会形成谋发展、快发展、大发展的良好氛围。

　　真抓实干，提升工作效能，增强执行力。大兴调查研究之风，市县两级政府带头精简会议、文件和事务性活动，集中精力深入基层、深入企业、深入群众，研究具体办法，解决实际问题，做到落实有力、服务到位、成效明显。全面推行政府绩效考核，围绕全年重点工作，逐项进行责任分解，强化督促检查，实行周调度、月检查、季通报，每个经济指标，每项工作任务，对人民群众的每件承诺，都要做到责任有主体、实施有监督、目标有考核，以政绩论英雄，年终看结果。

　　强化服务，优化发展环境，增强凝聚力。在政府系统强化全市“一盘棋”的思想，加强团结协作，形成坚强的工作合力，确保政令畅通。进一步转变政府职能，围绕项目建设开展服务、围绕全民创业开展服务、围绕招商引资开展服务，围绕重大民生问题开展服务。进一步规范和完善审批程序，压缩审批时限，强化对窗口单位的监管，推进政府服务提速增效。加大涉软案件惩处力度，严肃查处不作为、乱作为、推诿扯皮等突出问题，努力营造规范高效、诚实守信的发展环境。

　　清正廉洁，树立良好形象，增强战斗力。严格落实党风廉政建设责任制，坚持依法行政，用制度管权、管事、管人，确保权力规范运行。全面推进“阳光政务”，规范建设工程招投标、国债资金使用、土地征用出让、矿产资源开发、政府采购和国有资产转让等公共资源管理。加大纠风专项治理力度，继续开展商业贿赂专项整治和建设工程领域突出问题的专项治理。针对易发多发腐败问题的关键部门和实权岗位，注重从源头上防治，树立政府清正廉洁形象。

　　各位代表！新的一年，我们正处在抢抓机遇、乘势快上、加快发展的关键时期。全市上下要在省委、省政府和市委的正确领导下，解放思想，开拓创新，埋头苦干，扎实工作，一心一意谋发展，全心全意惠民生，竭尽全力保稳定，为推进白山经济社会又好又快发展而努力奋斗！
